# Norisoboldine Suppresses VEGF-Induced Endothelial Cell Migration via the cAMP-PKA-NF-κB/Notch1 Pathway

**DOI:** 10.1371/journal.pone.0081220

**Published:** 2013-12-09

**Authors:** Qian Lu, Bei Tong, Yubin Luo, Li Sha, Guixin Chou, Zhengtao Wang, Yufeng Xia, Yue Dai

**Affiliations:** 1 State Key Laboratory of Natural Medicines, Department of Pharmacology of Chinese Materia Medica, China Pharmaceutical University, Nanjing, China; 2 Department of Pediatrics, Nanjing Maternal and Child Health Hospital of Nanjing Medical University, Nanjing, China; 3 Institute of Chinese Materia Medica, Shanghai University of Traditional Chinese Medicine, Shanghai, China; University of Leuven, Rega Institute, Belgium

## Abstract

The migration of endothelial cells has been regarded as a potential target for the treatment of angiogenesis-related diseases. Previously, we demonstrated that norisoboldine (NOR), an alkaloid compound isolated from Radix Linderae, can significantly suppress synovial angiogenesis by selectively inhibiting endothelial cell migration. In this study, we evaluated the importance of various pathways in VEGF-induced endothelial cell migration using specific inhibitor. VEGF-induced endothelial cell migration and sprouting were significantly inhibited by H-89 (an inhibitor of protein kinase A (PKA)) but not by inhibitors of other pathways. NOR markedly suppressed VEGF-induced intracytoplasmic cAMP production and PKA activation and thereby down-regulated the activation of downstream components of the PKA pathway, including enzymes (src, VASP and eNOS) and the transcription factor NF-κB. Moreover, the transcription activation potential of NF-κB, which is related to IκBα phosphorylation and the disruption of the p65/IκBα complex, was reduced by NOR. Meanwhile, NOR selectively inhibited the expression of p-p65 (ser276) but not p-p65 (ser536) or PKAc, indicating that PKAc participates in the regulation of NF-κB by NOR. Co-immunoprecipitation and immunofluorescence assays confirmed that NOR inhibited the formation of the PKAc/p65 complex and thereby decreased p65 (ser276) phosphorylation to prevent p65 binding to DNA. Docking models indicated that the affinity of NOR for PKA was higher than that of the original PKA ligand. Moreover, the fact that H-89 improved Notch1 activation, but DAPT (an inhibitor of Notch) failed to affect PKA activation, suggested that PKA may act on upstream of Notch1. In conclusion, the inhibitory effects of NOR on endothelial cell migration can be attributed to its modulation of the PKA pathway, especially on the processes of p65/IκBα complex disruption and PKAc/p65 complex formation. These results suggest that NOR inhibit VEGF-induced endothelial cell migration via a cAMP-PKA-NF-κB/Notch1 signaling pathway.

## Introduction

Angiogenesis is defined as the generation of new vascular growth by sprouting from pre-existing vessels [1], [2]. Physiological angiogenesis is a vital mechanism during embryonic development and natural wound healing. In contrast, pathological angiogenesis is involved in the development of many diseases, including cancers, proliferative retinopathy and rheumatoid arthritis [1]–[3]. Angiogenesis is a multistep process that begins with the release of matrix-degrading enzymes and the migration of endothelial cells induced by angiogenic cytokines, such as vascular endothelial growth factor (VEGF), platelet derived growth factor (PDGF) and basic fibroblast growth factor (bFGF) [3]. Understanding the detailed mechanisms of endothelial cell migration and identifying inhibitors with better clinical efficacy and safety may be helpful in the development of anti-angiogenic agents.

Numerous endogenous factors are involved in the regulation of angiogenesis. VEGF, the most well-described pro-angiogenic factor, plays a crucial role in both physiological and pathological angiogenesis [4]. VEGF can be produced in response to many stimuli such as hypoxia and inflammation [4], [5]. The angiogenic effect of VEGF depends on binding to vascular endothelial growth factor receptor 2 (VEGFR_2_) in the endothelial cell membrane. VEGFR_2_, a receptor tyrosine kinase, can activate a series of downstream signaling pathways, including the p38 mitogen-activated protein kinase (MAPK), extracellular signal regulated kinase 1 and 2 (ERK1/2), Jun-amino-terminal kinase/stress-activated protein (JNK/SAPK), AKT, protein kinase C (PKC) and protein kinase A (PKA) pathways [4]–[10]. These pathways have been reported to participate in the regulation of angiogenesis and endothelial cell function, but which pathway is primarily responsible for endothelial migration still remains unclear.

Norisoboldine (NOR) is the primary isoquinoline alkaloid constituent of Radix Linderae, which is the dry roots of *Lindera aggregata* used in traditional Chinese medicine. Previously, we reported a beneficial effect of NOR on joint destruction in adjuvant-induced arthritis (AIA) rats and demonstrated that the inhibition of synovial angiogenesis contributed, at least partially, to the effects of NOR [11], [12]. NOR functions in a way that is distinct from other natural products such as curcumin; it has little effect on the proliferation of endothelial cells (human umbilical vein endothelial cells), but selectively suppresses the migration of endothelial cells by preventing the Notch1 pathway-related tip cell phenotype [11]. Its anti-migration activity was also confirmed in human microvascular endothelial cells at concentrations without significant cytotoxicity (data not shown).

In the present study, we investigated which pathway plays the key role in the regulation of VEGF-induced endothelial cell migration. Next, we clarified the effect of NOR on this pathway and explored the interplay between this pathway and the Notch1 pathway using specific inhibitors.

## Materials and Methods

### Reagents and Chemicals

**Figure 1:**
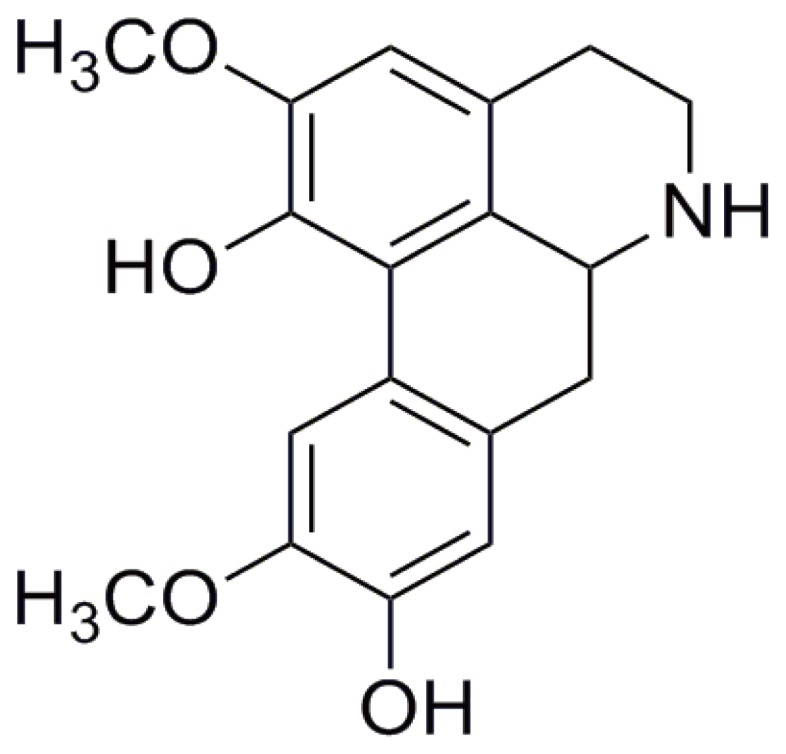
The structure of norisoboldine (NOR).

NOR (>98%) was prepared by Prof. Guixin Chou (Fig. 1). Its structure was identified by ^1^H- and ^13^C-nuclear magnetic resonance, and its purity was tested by high-performance liquid chromatography [11], [13]. Medium 199 (M199), endothelial cell growth medium-2 (EGM-2), trypsin, penicillin and streptomycin were purchased from Gibco BRL (Grand Island, NY, USA). Newborn calf serum (NBCS) was purchased from PAA Laboratories GmbH (Morningside, QLD, Australia). N-[N-(3, 5-difluorophenacetyl)-L-alanyl]-S-phenylglycine t-butyl ester (DAPT), curcumin, Tween-20, bovine serum albumin (BSA), sodium dodecyl sulfate (SDS) and dithiothreitol were purchased from Sigma Chemical (St Louis, MO, USA). SB203580 (a specific inhibitor of p38 MAPK), U0126 (a specific inhibitor of ERK), SP600125 (a specific inhibitor of JNK), triciribine (a specific inhibitor of protein kinase B (AKT/PKB)), chelerythrine (a specific inhibitor of protein kinase C (PKC)), H-89 (a specific inhibitor of PKA), the anti-p-p65 (ser276) antibody, the anti-p-p65 (ser536) antibody, the p-IκBα antibodies, the glyceraldehyde-3-phosphate dehydrogenase (GAPDH) monoclonal antibody, the enhanced chemiluminescent plus reagent kit and peroxidase-conjugated secondary antibodies were purchased from KangChen Bio-tech (Shanghai, China). Antibodies against P-PKA, p-src, p-vasodilator-stimulated phosphoprotein (VASP), p-endothelial nitric oxide synthase (eNOS), p65, cAMP response element binding protein (CREB) and the FITC- and TRITC-conjugated secondary antibodies were purchased from ImmunoWay Biotechnology (Newark, DE, USA). The antibody against the catalytic subunit of PKA (PKAc) was purchased from Santa Cruz Biotechnology (Santa Cruz, CA, USA). The anti-IκBα antibody was obtained from the Beyotime Institute of Biotechnology (Nantong, China). The antibody against cleaved Notch1 was obtained from Bioworld Technology (St. Louis Park, MN, USA). Recombinant human VEGF_165_ was obtained from Peprotech (Rocky Hill, NJ, USA). The other chemicals and reagents used were of analytical grade.

### Ethics Statements

Ethical approval for this research was obtained from the Human Research Ethics Committee of Nanjing Maternal and Child Health Hospital, Nanjing Medical University. Written informed consent was obtained from the mothers. All experiments were conducted in accordance with the ethical regulations and current laws of China.

### Isolation and Treatment of Human Umbilical Vein Endothelial Cells (HUVECs)

Umbilical cords were obtained from Nanjing Maternal and Child Health Hospital of Nanjing Medical University (Nanjing, China). Endothelial cells were isolated from the cords as described previously [14]. The cells were harvested and seeded into 25 cm^2^ flasks that were pre-coated with 0.2% (w/v) gelatin (Promega, Madison, WI, USA). The culture medium consisted of M199 supplemented with 20% (v/v) NBCS, 2 mM L-glutamine, 5 U/ml penicillin G, 5 µg/ml streptomycin sulfate and 7.5 µg/ml endothelial cell growth supplement (Sigma). The cells were cultured at 37°C in a humidified atmosphere containing 5% CO_2_. The medium was changed after 24 h and every two days thereafter until confluence was achieved. All of the experiments in this study were performed using HUVECs within passages 3–5.

### MTT and LDH Assays

Cell viability was evaluated using MTT assays. HUVECs were plated into 96-well plates at a density of 2×10^5^ cells/ml and cultured in a humidified 5% CO_2_-containing atmosphere at 37°C for 12 h. They were treated with different concentrations of NOR (1, 3, 10, 30, 60 and 100 µM) for 24 h. Aliquots of MTT (5 mg/ml, 20 µl/well) were added to each well, and the plates were incubated for an additional 4 h at 37°C. The supernatants were carefully removed, and DMSO (150 µl/well) was added into each well to dissolve the formazan crystals. The absorbance at 570 nm (A_570 nm_) was read using a Model 1500 Multiskan spectrum microplate reader (Thermo, Waltham, MA, U.S.A.).

For LDH assays, HUVECs were seeded into 96-well plates at a density of 2×10^5^ cells/ml. They were treated with the indicated concentrations of NOR for 24 h. After treatment with NOR, the cell supernatants were collected, and the levels of LDH were measured using ELISA kits according to the manufacturer’s instructions.

### Fibrin Bead Assay

This experiment was conducted as described previously [15], [16]. In brief, HUVECs were mixed with Cytodex 3 microcarriers (Amersham Pharmacia Biotech, Piscataway, NJ, USA) at a concentration of 400 HUVECs per bead in 1 ml EGM-2 (Gibco). The beads with cells were shaken gently every 20 min for 4 h at 37°C with 5% CO_2_ and then incubated in 5 ml EGM-2 for an additional 12–16 h. The beads were then re-suspended at a concentration of 200 cell-coated beads/ml in 2.5 mg/ml fibrinogen (Sigma) and 0.15 units/ml aprotinin (Sigma) and seeded in a 24-well plate (0.5 ml/well), and then 0.625 units of thrombin (Sigma) were added to the wells. Capillary-like sprouts from the beads appeared on day 2–3. Lumen formation began on approximately day 5–6. Over the course of 10–20 days, extensive networks were formed. The indicated drug treatments were added on day 3, and five random ×200 fields were photographed for each well on day 14. The number and length of sprouts were measured, averaged and compared.

### Reverse-Transcription Polymerase Chain Reaction Assay (RT-PCR)

RNA was extracted from cells and converted to cDNA as follows: HUVECs in the logarithmic growth phase were placed into six-well flat-bottom tissue culture plates at a density of 2×10^5^ cells/ml. Total RNA (0.5 mg) was isolated using Trizol reagent (Invitrogen, Carlsbad, CA, USA). Reverse transcription was performed using an oligo (dT)_18_ primer and M-MLV reverse transcriptase (Invitrogen) at 37°C for 50 min. The primer sequences were chosen as follows: hairy/enhancer-of-split related with YRPW motif (HEY) 1 primers, (sense) 5′-CGA GGT GGA GAA GGA GAG TG-3′ and (antisense) 5′-CTG GGT ACC AGC CTT CTC AG-3′; HEY2 primers, (sense) 5′-TTC AAG GCA GCT CGG TAA CT-3′ and (antisense) 5′-GGG CAT TTT ACT TCC CCA AT-3′; and β-actin primers, (sense) 5′-ACA TCT GCT GGA AGG TGG AC-3′ and (antisense) 5′-GGT ACC ACC ATG TAC CCA GG-3′. The thermal cycling programs were as follows: To amplify HEY1 and β-actin, the reactions were subjected to 32 cycles of 94°C for 1 min, at 55°C for 45 s and at 72°C for 1 min and to amplify HEY2, the reactions were subjected to 40 cycles of 94°C for 1 min, at 55°C for 1 min and at 72°C for 1 min. The amplified products were electrophoresed on a 1% agarose gel and visualized by GoldView™ (SBS Genetech, Beijing, China) and ultraviolet irradiation [17].

### Western Blot Assay

The levels of PKAc, p-PKA, p-src, p-VASP, p-eNOS, p65, p-p65 (ser276), p-p65 (ser536), IκBα, p-IκBα and Cleaved Notch1 protein were detected by western blot analysis as described previously [18]. GAPDH was used as an internal standard. HUVECs were treated with NOR or curcumin in the presence or absence of VEGF for 24 h. Total cellular protein was extracted with RIPA lysis buffer (SunShineBio, Nanjing, China) for 20 min on ice. The lysates were clarified by centrifugation at 12,000 rpm for 5 min at 4°C. The concentrations of protein in the supernatants were determined using bicinchoninic acid protein assay. Equal amounts of protein lysate were separated by 10% SDS-PAGE and then transferred to PVDF membranes. The membranes were blocked with 10% non-fat dry milk for one hour at room temperature and then incubated with antibodies overnight at 4°C. Subsequently, they were washed three times with PBS Tween-20 (PBST) buffer and incubated with secondary antibodies for one hour at room temperature. Immunoreactive bands were visualized using film exposure with enhanced chemiluminescence detection reagents.

### Coimmunoprecipitation Assay

HUVECs were treated with NOR or curcumin in the presence of VEGF for 24 h. Next, cells were lysed with coimmunoprecipitation lysis buffer (SunShineBio). Cell lysates (containing 800 mg protein) were incubated with 0.2–2 µg of the indicated antibodies overnight at 4°C and then with 20 µl Protein A/G Plus-Agarose (Beyotime) for another 4 h at 4°C [19], [20]. The beads were then washed three times with coimmunoprecipitation lysis buffer, resolved by 10% SDS-PAGE, and immunoblotted with antibodies against various proteins.

### Immunofluorescence Assay

HUVECs were treated with NOR or curcumin in the presence of VEGF for 24 h. Immunofluorescence assays were performed as described previously [20], [21]. Cells were fixed with 4% paraformaldehyde for 30 min, incubated with PBS for 15 min, permeabilized with 1% Triton X-100, and blocked with 2% BSA for 30 min. The cells were then incubated with 100 µl of primary antibodies against PKAc and p65 overnight at 4°C. After washing, the cells were exposed to FITC and TRITC-conjugated secondary antibodies. Images were observed and captured using an Olympus IX51 fluorescence microscope (Tokyo, Japan).

### Electrophoretic Mobility Shift Assay (EMSA)

The DNA-binding activities of NF-κB and CREB were confirmed with biotin-labeled oligonucleotide probes (5′-AGT TGA GGG GAC TTT CCC AGG C-3′; 3′-TCA ACT CCC CTG AAA GGG TCC G-5′) and (5′-AGA GAT TGC CTG ACG TCA GAG AGC TAG-3′; 3′-TCT CTA ACG GAC TGC AGT CTC TCG ATC-5′), respectively. Nuclear extracts from HUVECs were prepared using a Nucleoprotein Extraction Kit (Sangon Biotech, Shanghai, China). The nuclear extracts mixed with probe were incubated with 1 µg/µl of poly (deoxyinosinic-deoxycytidylic) acid in binding buffer for 30 min at 4°C, resolved on a 6.5% polyacrylamide gel at 100 V for 1 h, and then transferred to a nylon membrane. For supershift assays, the nuclear extracts from VEGF-treated cells were incubated with p65 or CREB antibodies for 30 minutes at 37°C before the complexes were analyzed by EMSA. The immunoreactive bands on nylon membranes were visualized by film exposure with enhanced chemiluminescence detection reagents [22].

### Wound Healing Assay

HUVECs were cultured in a six-well plate at a density of 2×10^5^ cells/ml. When the cells reached 90% confluence, a single wound was created in the center of the cell monolayer by scratching with a sterile plastic pipette tip. The debris was removed by washing with serum-free medium. The cells were then treated with specific pathway inhibitors and VEGF (10 ng/ml) for 10 h. The wound area was estimated using a light microscope (Olympus IX51, Japan) equipped with a digital camera, and three measurements of wound area were made at randomly chosen points. The extent of wound closure was presented as the percentage by which the original scratch area had decreased at each measured time point [23].

### Measurements of cAMP Level and PKA Activity

HUVECs were seeded into 96-well plates at a density of 2×10^5^ cells/ml. The cells were treated with different concentrations of NOR or curcumin in the presence of VEGF for 24 h. After treatment, cell lysates were collected, and the levels of cAMP and activity of PKA were measured using ELISA kits according to the manufacturer’s instructions.

### Identification of a Potential Target of NOR in the PKA Pathway [24], [25]

To further understand how NOR affects the PKA pathway, a preliminary in silico study was performed to identify potential targets. AutoDock4.2 software is commonly used for these analyses due to its automated docking capability. In this study, the 3D structure of NOR was generated and minimized in the MMFF94 force field by Discovery Studio, and the 2D structures of NOR were constructed and hydrogen atoms were added using ChemOffice software. We then used the protein-ligand complex crystal structures of PKA from the RCSB Protein Data Bank (http://www.rcsb.org/pdb/home/home.do) and docked NOR with PKA in AutoDock4.2. The original ligand in the complex structures was used as a reference compound to judge the affinity of NOR for the PKA target. The binding site was defined as a 40×40×40 Å cube centered on the occupied space of the original ligand with a spacing of 0.375 Å between the grid points.

### Statistical Analysis

The data are expressed as the mean ± SD of three independent experiments. Comparisons between multiple groups were performed using one-way analysis of variance and Dunnett’s test. Values of p<0.05 were considered to be statistically significant.

## Results

### The Effect of NOR on the Viability of HUVECs

**Figure 2:**
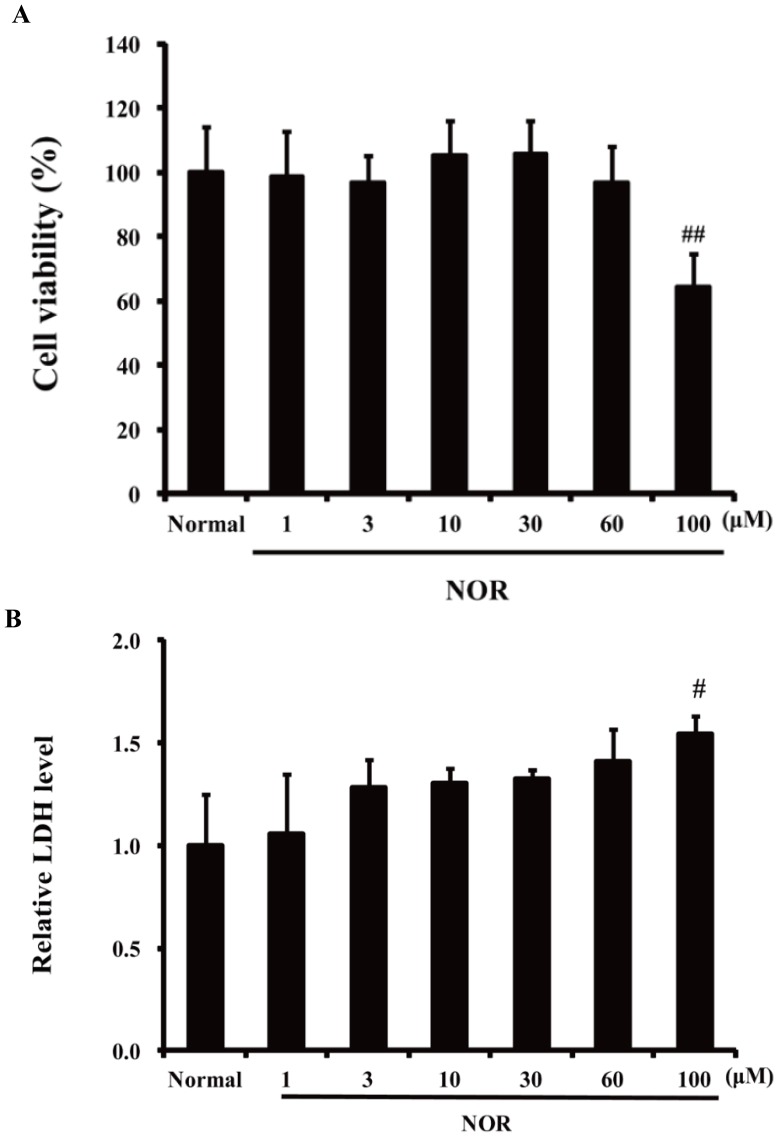
Cytotoxicity of norisoboldine (NOR) against HUVECs. (**A**) MTT assay. HUVECs seeded in 96-well plate were incubated with NOR (1, 3, 10, 30, 60, 100 µM) for 24 h. Then MTT assay was used to evaluate the viability. (**B**) LDH assay. HUVECs seeded in 96-well plate were incubated with NOR (1, 3, 10, 30, 60, 100 µM) for 24 h. Relative LDH release was used as an index of cytotoxicity. The data were expressed as mean ± SD of three independent experiments. ^#^ *P*<0.05,^ ##^ *P*<0.01, *vs*. normal group.

To rule out the possibility that NOR was cytotoxic in HUVECs at the test concentrations, MTT and LDH assays were performed. As shown in Fig. 2, treatment with NOR (1, 3, 10, 30, and 60 µM) for 24 h has no effect on the cell viability of HUVECs. These concentrations were therefore used in the following experiments.

### The Role of the PKA Pathway in VEGF-induced Endothelial Cell Migration

**Figure 3:**
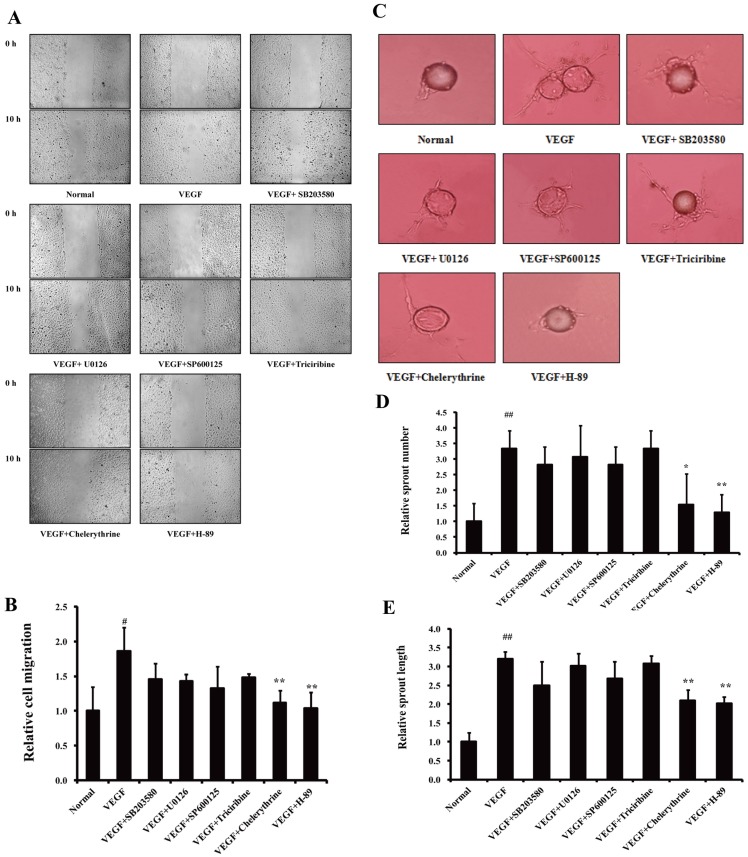
The role of the PKA pathway in the VEGF-induced endothelial cell migration. (**A**). Effects of specific inhibitors of various pathways on wound healing. Scratch wounds were created in cell monolayers of HUVECs using a sterile pipette tip. Then cells were cultured with inhibitors (10 µM) in the presence of VEGF (10 ng/ml) for 10 h. (**B**). Quantification of wound area (The initial wound area minus wound area after 10 h). (**C**). Effects of specific inhibitors of various pathways on VEGF-induced sprouting of endothelial-cell-coated beads in fibrin gel. Cells on beads were exposed to different treatments, and photographs were taken on day 14. (**D**). Quantification of sprout number. (**E**). Quantification of sprout length. The data are expressed as mean ± SD of three independent experiments. ^#^ *P*<0.05, ^##^ *P*<0.01 *vs*. normal group; *P<0.05, **P<0.01 *vs*. VEGF group. (original magnification: ×100 in A, ×200 in B).

It has been reported that multiple pathways, including the p38 MAPK, ERK1/2, JNK/SAPK, AKT/PKB, PKC and PKA pathways, may participate in the regulation of angiogenesis induced by VEGF [4]–[10]. To clarify the anti-migration mechanisms of NOR, specific inhibitors of these pathways were used to determine which pathway plays a key role in VEGF-induced endothelial cell migration. Wound healing assays were used to examine cell migration. As shown in Fig. 3A and B, H-89 (a PKA inhibitor, 10 µM) and chelerythrine (a PKC inhibitor, 10 µM) reversed the VEGF-induced migration of HUVECs. The fibrin bead assay is a novel and specific method to evaluate the migration ability of endothelial cells with a focus on endothelial sprouting. Compared to the inhibitors of other pathways, H-89 (10 µM) showed strongest inhibitory effect on both sprout number and length (Fig. 3C to E). These results indicated that the PKA pathway plays a key role in VEGF-induced endothelial cell migration.

### The Effect of NOR on the VEGF-induced Intracytoplasmic cAMP Level and PKA Activation in HUVECs

**Figure 4:**
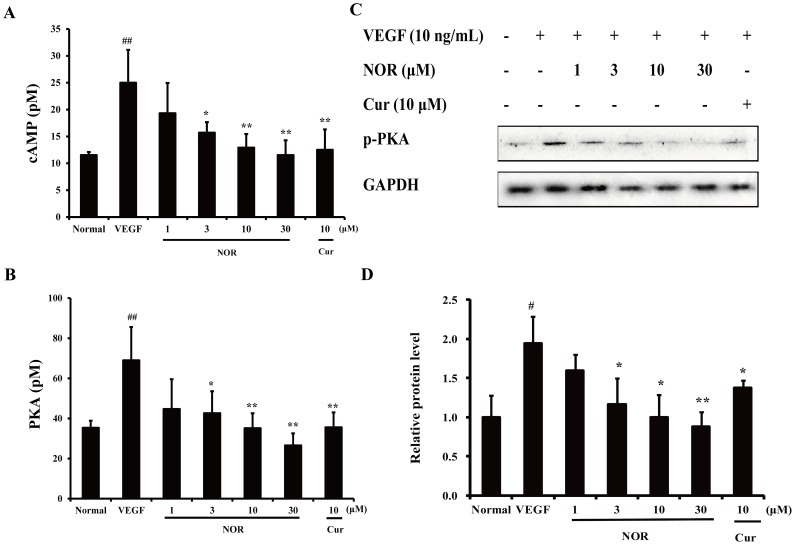
The effect of NOR on the VEGF-induced intracytoplasmic cAMP level and PKA activation in HUVECs. (**A**). Effect of NOR on VEGF-induced intracytoplasmic cAMP level. Cells were treated with NOR and VEGF (10 ng/ml) for 24 h. (**B**). Effect of NOR on VEGF-induced PKA catalytic activity. Cells were treated with NOR and VEGF (10 ng/ml) for 24 h. (**C**). Effect of NOR on VEGF-induced PKA phosphorylation. Cells were pretreated with NOR for 24 h, stimulated by VEGF for 20 min, and then harvested for western blot analysis. (**D**). Quantification of PKA phosphorylation. The data are expressed as mean ± SD of three independent experiments. ^#^ *P*<0.05, ^##^ *P*<0.01 *vs*. normal group; *P<0.05, **P<0.01 *vs.* VEGF group. NOR, norisoboldine; Cur, curcumin.

Previous studies have demonstrated that NOR can inhibit VEGF-induced endothelial cell migration [11], [12]. To clarify whether this effect is related to the inhibition of the PKA pathway, the intracytoplasmic cAMP level and PKA activation in HUVECs were investigated. As shown in Fig. 4A, VEGF stimulation for 24 h led to a significant elevation of the cytoplasmic cAMP level in HUVECs. Treatment with NOR (1, 3, 10, and 30 µM) resulted in a concentration-dependent inhibition of cAMP levels, with inhibitory percentages of 22.8%, 37.3%, 48.4% and 53.9%, respectively. Notably, NOR (30 µM) almost completely abrogated the production of cAMP caused by VEGF. The activation of PKA mostly depends on the intracytoplasmic cAMP level [26]. Both the catalytic activity and phosphorylation of PKA were inhibited by NOR (Fig. 4B to D). Notably, NOR (10, 30 µM) almost completely abrogated PKA activity. These data indicated that the effect of NOR on endothelial cell migration can be attributed to its inhibition of VEGF-induced cAMP production and PKA activation.

### The Effect of NOR on the VEGF-induced Activation of Various Kinases in the PKA Pathway

**Figure 5:**
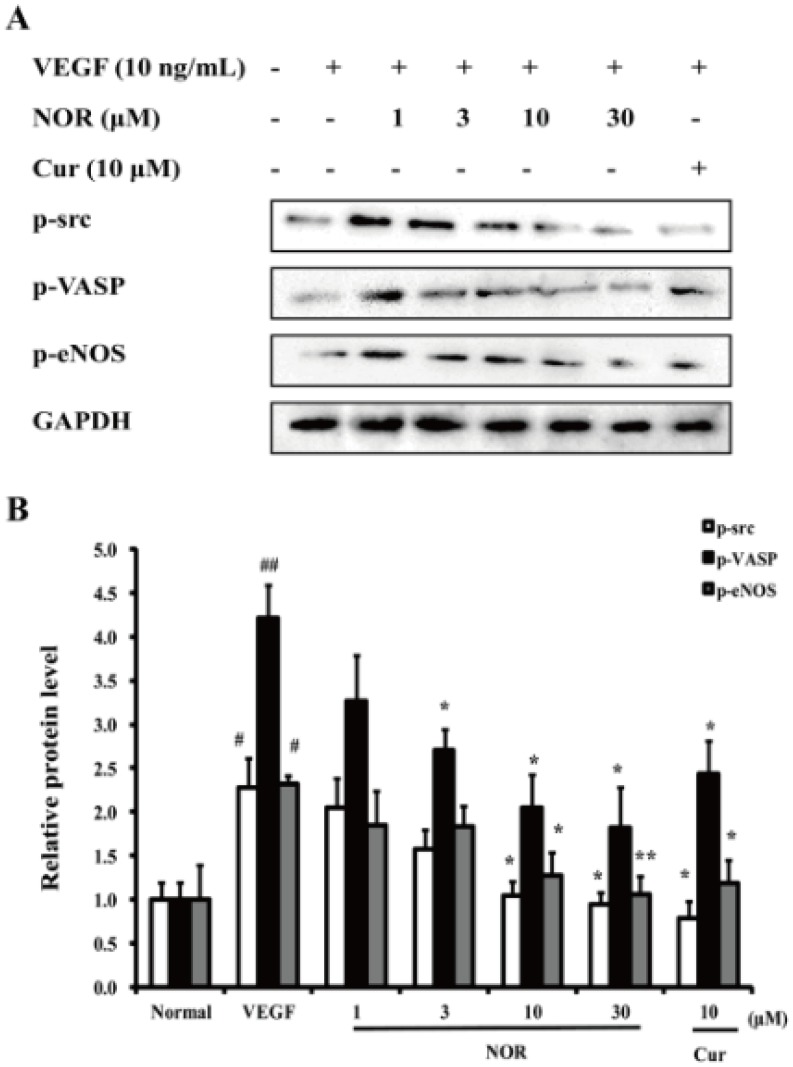
The effect of NOR on the VEGF-induced activation of various kinases in the PKA pathway. (**A**). Effect of NOR on VEGF-induced expressions of p-src, p-VASP and p-eNOS. HUVECs were pretreated with NOR for 24 h, stimulated by VEGF for 20 min, and then harvested for western blot analysis. (**B**) Quantification of p-src, p-VASP and p-eNOS expressions. The data were expressed as mean ± SD of three independent experiments. ^#^ *P*<0.05, ^##^ *P*<0.01 *vs*. normal group; *P<0.05, **P<0.01 *vs*. VEGF group. NOR, norisoboldine; Cur, curcumin.

src, VASP and eNOS are important enzymes that are activated by PKA and play critical roles in the processes of endothelial cell migration [27]–[29]. As shown in Fig. 5A and B, p-src, p-VASP and p-eNOS protein levels were moderately increased after VEGF stimulation. Treatment with various concentrations of NOR (1, 3, 10, and 30 µM) significantly decreased the levels of these three proteins. Curcumin (10 µM) also markedly inhibited the VEGF-induced expression of p-src, p-VASP and p-eNOS. Notably, NOR (10 and 30 µM) and curcumin (10 µM) almost reversed the increased phosphorylation of src. These findings suggested that NOR could down-regulate the VEGF-induced activation of various kinases in the PKA pathway.

### The Effect of NOR on the VEGF-induced Activation of Transcription Factors in the PKA Pathway

**Figure 6:**
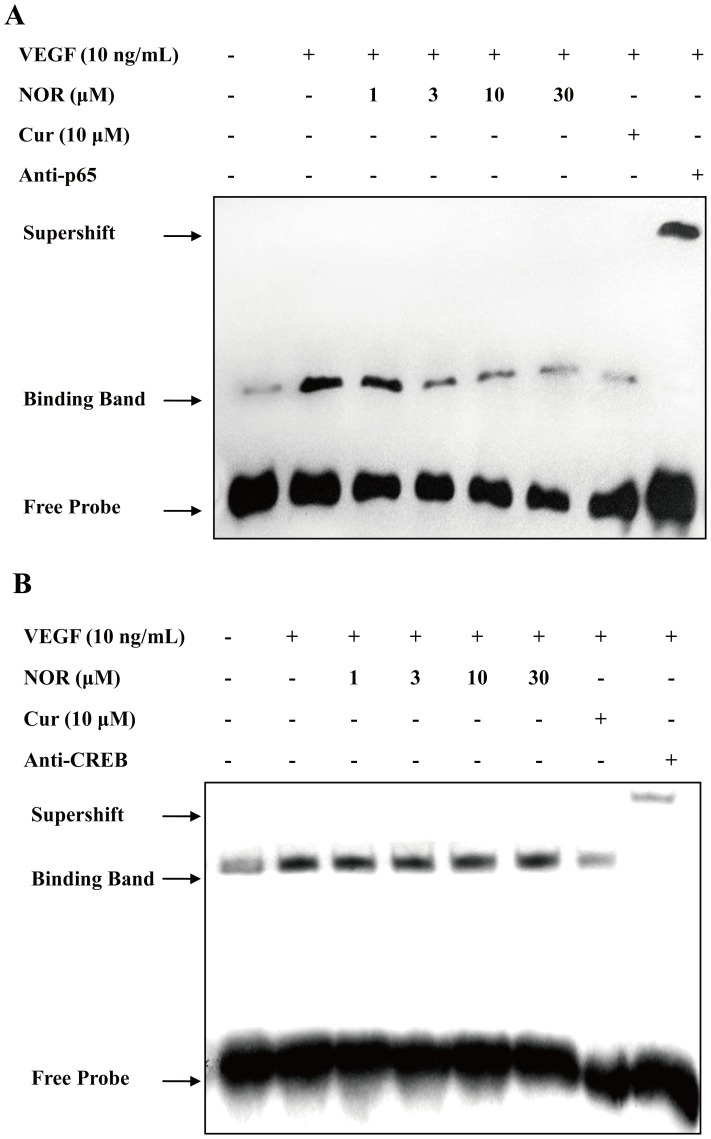
The effect of NOR on the VEGF-induced activation of transcription factors in the PKA pathway. (**A**). Effect of NOR on VEGF-induced NFκB activation. HUVECs were treated with NOR or Cur in the presence of VEGF for 24 h, and then harvested for EMSA analysis. (**B**). Effect of NOR on VEGF-induced CREB activation. HUVECs were treated with NOR or Cur in the presence of VEGF for 24 h, and then harvested for EMSA analysis. NOR, norisoboldine; Cur, curcumin.

NF-κB and CREB are nuclear factors that can be activated by PKA [30], [31]. They are also reported to regulate the transcription of many migration-related genes [32], [33]. As shown in Fig. 6A and B, the transcription activity of NF-κB and CREB was moderately increased after VEGF stimulation. Treatment with NOR (1, 3, 10, and 30 µM) significantly decreased NF-κB activation but failed to affect CREB activation. Treatment with curcumin (10 µM) markedly inhibited the transcription activity of both NF-κB and CREB. These findings suggested that NOR, in contrast to curcumin, selectively down-regulated the VEGF-induced activation of NF-κB but not CREB.

### The Effect of NOR on the VEGF-induced Activation of the NF-κB Pathway

**Figure 7:**
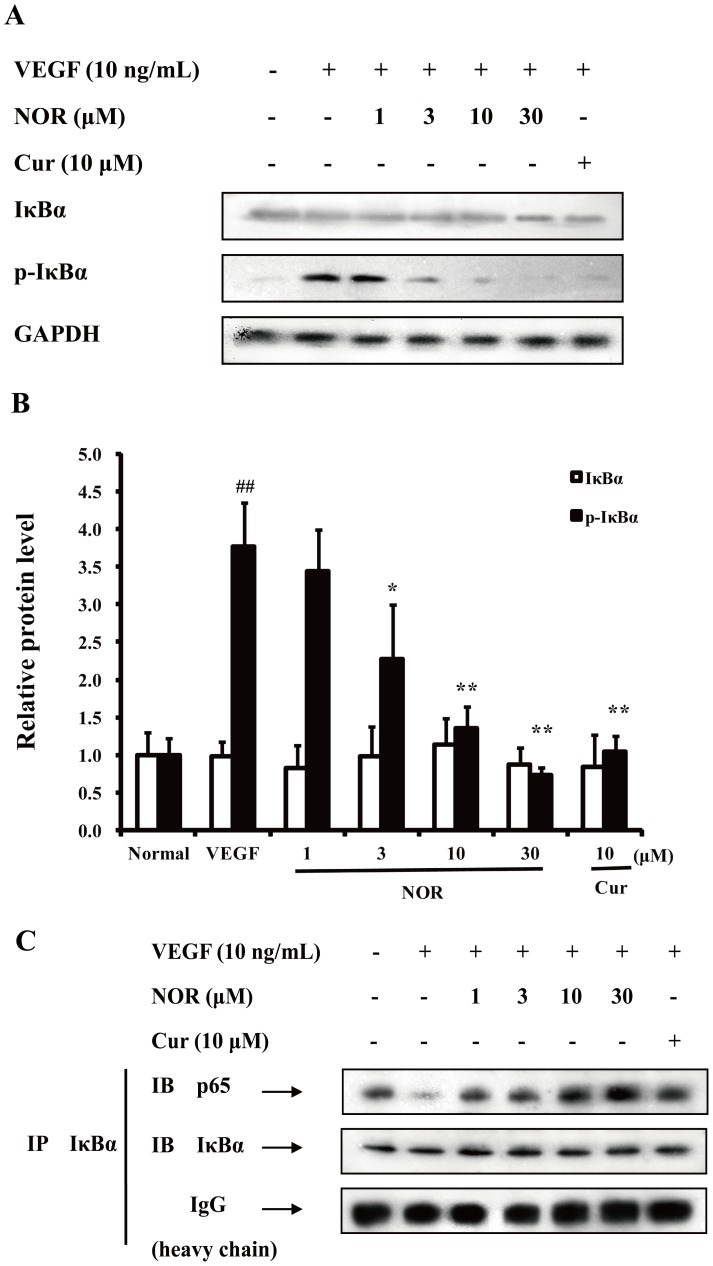
The effect of NOR on the VEGF-induced activation of the NF-κB pathway. (**A**). Effect of NOR on VEGF-induced expressions of IκBα and p-IκBα in HUVECs. (**B**). Quantification of IκBα and p-IκBα expressions. (**C**). Effect of NOR on the disruption of p65/IκB complex in VEGF-activated HUVECs. Cells were treated with NOR or Cur in the presence of VEGF for 24 h. Then cell lysates were immunoprecipitated with IκBα antibody and immunoblotted with p65 antibody. The data were expressed as mean ± SD of three independent experiments. ^##^ *P*<0.01 *vs*. normal group; *P<0.05, **P<0.01 *vs.* VEGF group. NOR, norisoboldine; Cur, curcumin.

P65 is one of the active components of NF-κB. During NF-κB activation, whether p65 can enter the nucleus depends on proteolytic degradation of IκB protein and disruption of p65/IκB complex [34]. Fig. 7A and B showed that VEGF (10 ng/ml) stimulation resulted in the noticeable phosphorylation of IκBα, which led to IκBα degradation. Treatment with NOR (1, 3, 10, and 30 µM) reduced IκBα phosphorylation in a concentration-dependent manner. Furthermore, NOR (30 µM) reversed the VEGF-increased phosphorylation of IκBα. The disruption of the p65/IκB complex was examined using coimmunoprecipitation assays. Cell lysates were immunoprecipitated with an anti-IκBα antibody and immunoblotted with an anti-p65 antibody. As shown in Fig. 7C, treatment with VEGF (10 ng/ml) decreased the association between p65 and IκBα and thereby caused the release of p65 from the complex. Treatment with NOR (1, 3, 10, and 30 µM) inhibited the disruption of the p65/IκB complex and prevented release of p65, indicating that the activation of p65 was suppressed by NOR. These findings suggested that the effect of NOR on the NF-κB pathway is related to its regulation of IκBα phosphorylation and subsequent inhibition of the disruption of the p65/IκB complex.

### The Effect of NOR on the Regulation of the NF-κB Pathway by PKA

**Figure 8:**
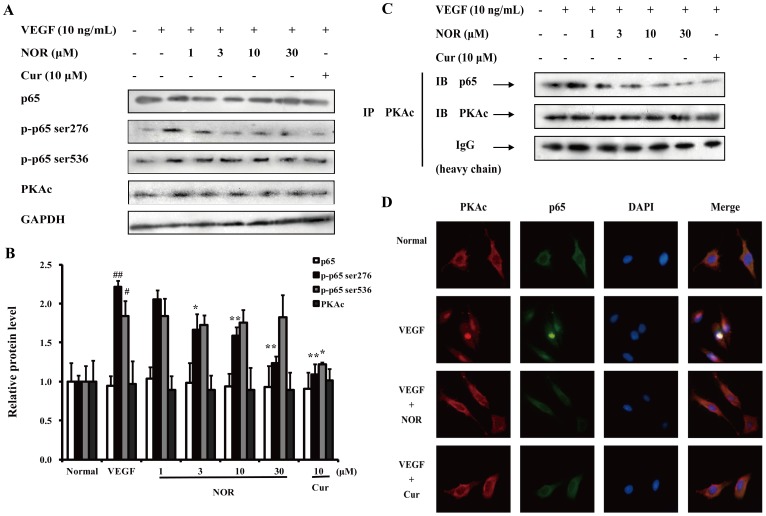
The effect of NOR on the participation of PKA in the regulation of the NF-κB pathway. (**A**). Effect of NOR on VEGF-induced expressions of p65, p-p65 (ser276), p-p65 (ser536) and PKAc in HUVECs. (**B**) Quantification of expressions of p65, p-p65 (ser276), p-p65 (ser536) and PKAc. (**C**). Effect of NOR on formation of PKAc/p65 complex in VEGF-activated HUVECs. Cells were treated with NOR or Cur in the presence of VEGF for 24 h. Then cell lysates were first immunoprecipitated with PKAc antibody and then immunoblotted with p65 antibody. (**D**). Effect of NOR on the co-localization of PKAc and p65 in HUVECs. Cells were cultured with NOR (10 µM) or Cur (10 µM) in the presence of VEGF (10 ng/ml) for 24 h. Then cells were immunofluorescence stained with PKAc and p65 antibodies. The data were expressed as mean ± SD of three independent experiments. ^#^ *P*<0.05, ^##^ *P*<0.01 *vs*. normal group; *P<0.05, **P<0.01 *vs*. VEGF group. NOR, norisoboldine; Cur, curcumin.

Previous reports suggested that PKA primarily phosphorylated serine 276 of p65 and thereby promoted the DNA binding activity and transcriptional activation potential of NF-κB [30]. As shown in Fig. 8A and B, VEGF (10 ng/ml) stimulation resulted in evident phosphorylation of both p65 (ser276) and p65 (ser536). Curcumin (10 µM) inhibited the phosphorylation of both sites of p65. However, NOR (1, 3, 10, and 30 µM) significantly reduced the levels of p-p65 (ser276) but did not affect the levels of p-p65 (ser536) and PKAc.

During the phosphorylation and nuclear translocation of p65 caused by PKA, PKAc and p65 form a PKAc/p65 complex [35]. In the present study, the formation of the PKAc/p65 complex was also examined using coimmunoprecipitation assays. Cell lysates were immunoprecipitated with an anti-PKAc antibody and immunoblotted with an anti-p65 antibody. As shown in Fig. 8C, VEGF (10 ng/ml) stimulation increased the association between PKAc and p65 and thereby improved the nuclear translocation and DNA binding activity of p65. Treatment with NOR (1, 3, 10, and 30 µM) inhibited the formation of the PKAc/p65 complex and suppressed p65 activation. The co-localization of PKAc and p65 in HUVECs was examined using immunofluorescence assays. As shown in Fig. 8D, VEGF promoted the association of PKAc and p65 and maintained the complex in nucleus. Both NOR and curcumin prevented the nuclear translocation of the PKAc/p65 complex. These findings indicated that the effect of NOR on NF-κB activation could be attributed to its modulation of PKA.

### *In silico* Docking of PKA and Binding Residues that Interact with NOR

**Figure 9:**
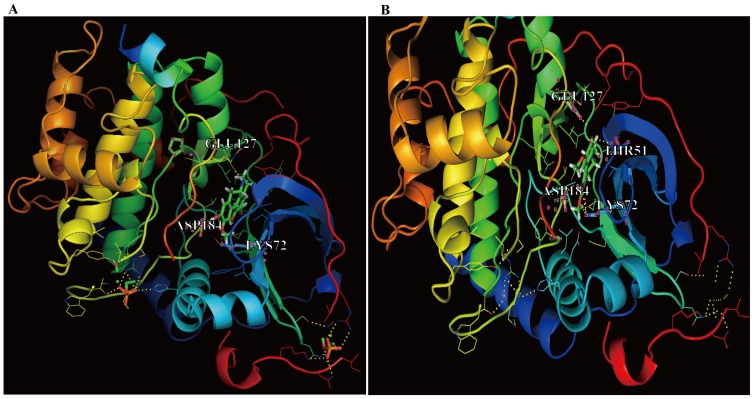
Computational structure prediction for PKA and docking simulations with NOR. Docking simulation results between the 3D structure of PKA and the chemical structures of (A) NOR-S (viridis color) and (B) NOR-R (viridis color). Docking simulations were performed using AutoDock4.2. The docking score of NOR-S and NOR-R were 5.06 and 4.96, respectively. The yellow dotted lines represent possible hydrogen bonds. GLU127, ASP184, LYS72 of PKA were predicted to hydrogen bond to NOR-S, and THR51, GLU127, ASP184, LYS72 of PKA were to NOR-R.

The docking simulation using AutoDock4.2 produced significant scores. The docking score between a given ligand and receptor is represented by various energy values such as electrostatic and van der Waals energy values and solvation energy. The docking scores for NOR-S, NOR-R and the original ligand of PKA were 5.06, 4.96 and 4.61, respectively. The results suggested that the binding affinity of NOR-S was greater than that of NOR-R. Using AutoDock4.2, we searched for binding residues within PKA that would lie close to the PKA ligand. As shown in Fig. 9, NOR-S was found to hydrogen bond with the GLU127, ASP184, and LYS72 residues of PKA, and NOR-R was found to hydrogen bond with the THR51, GLU127, ASP184, and LYS72 residues of PKA.

### Interplay between the PKA and Notch1 Pathways

**Figure 10:**
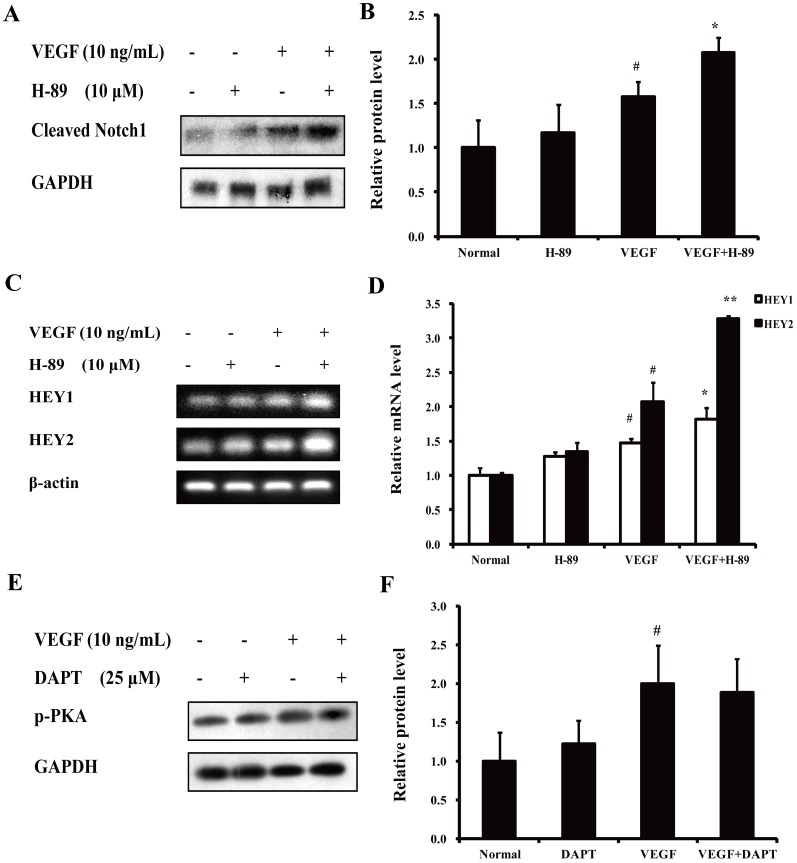
Interplay between PKA pathway and Notch1 pathway. (**A**). Effect of H-89 on VEGF-induced expression of Cleaved Notch1. (**B**). Quantification of Cleaved Notch1 expression. (**C**). Effect of H-89 on VEGF-induced HEY1 and HEY2 gene expressions. (**D**). Quantification of HEY1 and HEY2 gene expressions. (**E**). Effect of DAPT on VEGF-induced phosphorylation of PKA. (**F**). Quantification of PKA phosphorylation. The data were expressed as mean ± SD of three independent experiments. ^#^ *P*<0.05 *vs*. normal group; *P<0.05, **P<0.01 *vs.* VEGF group. NOR, norisoboldine; Cur, curcumin.

**Figure 11:**
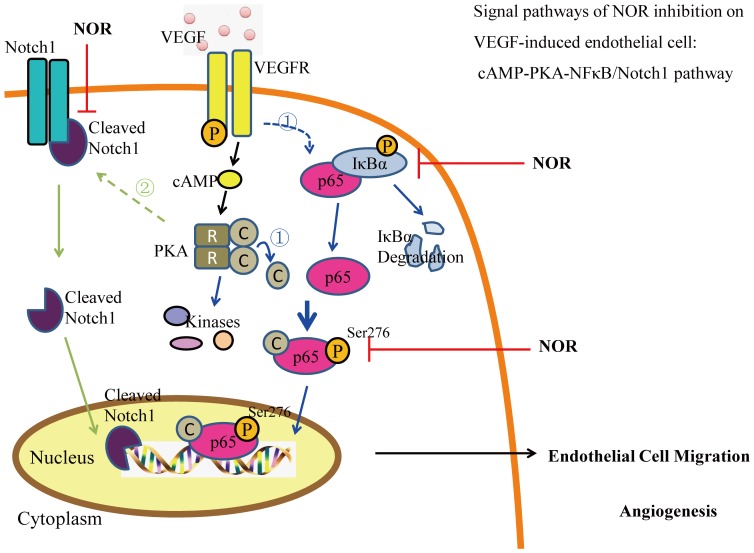
Signal pathways by which NOR inhibits the VEGF-induced endothelial cell migration: cAMP-PKA-NF-κB/Notch1 pathway. In details, NOR markedly suppressed VEGF-induced intracytoplasmic cAMP production and thereby PKA activation. This action, on one hand, inhibited IκBα degradation and p65/IκBα complex disruption, and then restrained PKAc/p65 complex formation and p65(ser276) phosphorylation to limit transcriptional activation potential of p65; on the other hand, promoted VEGF-induced the release of Notch1 active domain-Cleaved Notch1 to improve expressions of Notch1 targeted genes. NOR, norisoboldine.

In our previous studies, the anti-migration activity of NOR was partially attributed to the prevention of the Notch1 pathway-related tip cell phenotype [11], [12]. In this study, preliminary experiments were conducted to examine the interplay between the PKA and Notch1 pathways. As shown in Fig. 10A and B, the PKA inhibitor H-89 (10 µM) promoted the VEGF-induced expression of both the Notch1 active domain (Cleaved Notch1) and two Notch1 pathway-related genes (HEY1 and HEY2). However, the Notch inhibitor DAPT (10 µM) had little effect on the phosphorylation of PKA (Fig. 10C). Taken together, these results suggest that the signaling pathways that participate in the inhibitory effect of NOR on VEGF-induced endothelial cell migration can be ranked as follows: cAMP-PKA-NF-κB/Notch1 (Fig. 11).

## Discussion

There are many reports that suggest that multiple pathways participate in the VEGF-induced migration and proliferation of endothelial cells [4]–[10]. In previous studies, we revealed that NOR can inhibit the migration of HUVECs [11]. To clarify the anti-migration mechanisms of NOR, the p38 MAPK inhibitor SB203580, the ERK1/2 inhibitor U0126, the JNK/SAPK inhibitor SP600125, the AKT inhibitor triciribine, the PKC inhibitor chelerythrine and the PKA inhibitor H-89 were screened to evaluate the effects of these pathways on endothelial migration triggered by VEGF. H-89 and Chelerythrine showed significant migration inhibitory activity compared to the other inhibitors. This finding suggested that the PKA and PKC pathways may be more important in VEGF-induced endothelial cell migration, although the participation of the MAPK pathway cannot completely be ruled out. Notably, many reports have noted that the PKC pathway was responsible for the regulation of cell proliferation, survival and apoptosis; these studies indicated that the inhibition of endothelial cell migration by chelerythrine (10 µM) was due largely to the effect of chelerythrine on cell number [36]–[39]. Therefore, the regulation of the PKA pathway on endothelial cell migration appeared to be more direct and important.

PKA is one of the best-characterized members of the cAMP-dependent protein kinase superfamily [40]. In the inactive state, PKA exists as a tetramer consisting of two regulatory (R) and two catalytic (C) subunits. R subunits have two cAMP-binding sites (CBD). cAMP binding to the CBD promotes a conformational change in PKA, which initiates the dissociation of C subunits from R subunits. The C subunits then become catalytically active and can phosphorylate serine and threonine residues on target proteins, which are usually considered to be the kinase substrates of PKA. To determine whether the PKA pathway was involved in regulating the effect of NOR on endothelial migration, a series of experiments examining PKA activity were conducted. It was shown that NOR was able to reduce the intracytoplasmic cAMP production and PKA activation caused by VEGF, suggesting that the effect of NOR on endothelial cell migration can be attributed to its inhibition of the cAMP-PKA pathway.

The PKA pathway contains multiple migration-related enzymes such as src, VASP and eNOS. src is a non-receptor tyrosine kinase that can be activated by other kinases, such as PKA, AKT and FAK [27], [41]. Activated src has an influence on the development of the metastatic phenotype and is considered to be a critical component of the signal transduction pathways that control the functions of cancer cells [42]. VASP is an important substrate of PKA that regulates actin filament assembly and gathering and thereby participates in various cell behaviors related to the actin cytoskeleton such as motility, adhesion and contraction [28], [43]. Nitric oxide (NO), mainly generated by eNOS activation, has been shown to play a central role in ischemic neovascularization. Experimental evidence indicates that PKA is an important upstream signaling regulator leading to eNOS activation during ischemic neovascularization [29]. To clarify the impact of NOR on the cAMP-PKA pathway, we examined the phosphorylation of src, VASP and eNOS. NOR was found to reduce the activation of src, VASP and eNOS caused by VEGF, supporting the conclusion that the inhibitory effect of NOR on cell migration is related to its effect on the cAMP-PKA pathway.

The PKA pathway can activate several migration-related nuclear transcription factors such as NF-κB and CREB. NF-κB is involved in the rapid response to various stimuli such as hypoxia, oxidant stress and viral infections [30]. The activation of NF-κB leads to the transient regulation of a number of genes encoding cytokines, growth factors, immunomodulatory molecules and apoptosis-related factors [34]. CREB belongs to the cAMP-response element binding protein family. After CREB is phosphorylated at serine 133 by PKA, it binds to cAMP response elements and stimulates transcription [31]. To further clarify the impact of NOR on the cAMP-PKA pathway, EMSA tests were performed. The results showed that both NF-κB and CREB were activated by VEGF, indicating that NF-κB and CREB participate in VEGF-induced cell migration. Meanwhile, curcumin, the positive control compound, markedly inhibited the activation of both NF-κB and CREB. These results were in good agreement with previous work [44], [45]. However, NOR exhibited a concentration-dependent inhibitory effect on the transcriptional activity of NF-κB but not CREB. The selective action of NOR on NF-κB activation indicated that the NF-κB pathway may play an important role in mediating the effect of NOR on cell migration. Given this, the subsequent studies were carried out to understand the modulation of NF-κB transcriptional activity by NOR.

NF-κB proteins are a family of transcription factors that include five proteins, p50, p52, p65 (RelA), RelB and c-Rel. In cytoplasm, they exist as homo- and hetero-dimers. The most common dimer is the p65–p50 complex [30]. To exert a transcriptional effect, p65 must translocate to the nucleus and bind to DNA. In unstimulated cells, NF-κB is bound to IκB and held in an inactive from. IκB maintains p65 in the cytosol by masking the nuclear localization sequences of NF-κB. In the classical pathway triggered by cytokines or growth factors, IκB is phosphorylated by activated IκB kinases (IKK) and subsequently degraded. Therefore, p65 is released from the complex and is able to translocate to the nucleus. In nucleus, the binding between p65 and DNA depends on the phosphorylation of p65 [30], [34], [35]. Numerous pathways with the ability to regulate the transcriptional activation potential of p65 via phosphorylation have been identified. Ghosh S. et al have demonstrated that PKAc was contained in the NF-κB/IκB complex; PKAc was therefore one of the first kinases to be associated with the phosphorylation of p65 [35]. To enhance the transcriptional activity of p65, PKAc selectively phosphorylates serine 276 of p65 [30], [35] and translocates to the nucleus with p65 [21], [35]. Therefore, the disruption of the p65/IκBα complex and the formation of the PKAc/p65 complex are two main processes in the transcriptional activation of the PKA-NF-κB pathway.

To examine the disruption of the p65/IκBα complex, the effect of NOR on IκBα phosphorylation was first investigated. The results showed that NOR reduced the phosphorylation of IκBα but did not affect the protein expression of IκBα. Coimmunoprecipitation assay also revealed that NOR prevented the VEGF-induced disruption of the p65/IκBα complex, suggesting that NOR can inhibit NF-κB transcriptional activation by regulating the p65/IκBα complex. To evaluate the formation of the PKAc/p65 complex, the effect of NOR on the expression of p-p65 (ser276), p-p65 (ser536), p65 and PKAc was investigated. It was observed that NOR inhibited VEGF-induced p65 (ser276) phosphorylation. However, p-p65 (ser536), p65 and PKAc protein levels were not influenced by NOR, indicating that the regulation of NOR on p65 (ser276) is selective; this residue has also been reported to be selectively phosphorylated by PKAc [30], [35]. Given that NOR can influence the activation of PKA pathway but did not affect PKAc protein levels, we hypothesized that NOR might mediate the complex of PKAc and p65. This was first shown using coimmunoprecipitation assays. NOR treatment decreased the ability of PKAc to bind to p65. This may be the primary reason for the inhibitory effect of NOR on VEGF-induced p65 (ser276) phosphorylation. Furthermore, the immunofluorescence assay offered lively and vivid information about the distribution of PKAc and p65 and their association. NOR indeed prevented both the formation of the PKAc/p65 complex and the translocation of p65 to the nucleus. In conclusion, the regulation of NF-κB by NOR can be summarized as follows: On one hand, NOR inhibits the disruption of the p65/IκBα complex to keep p65 in the cytoplasm, and on the other hand, NOR inhibits the formation of the PKAc/p65 complex and thereby decreases p65 (ser276) phosphorylation to prevent p65 binding to DNA.

A docking simulation using AutoDock4.2 suggested that a direct interaction between PKA and NOR-S and NOR-R occurs at the active site. Furthermore, the docking scores between PKA and NOR-S/NOR-R (5.06/4.96) calculated by the docking program were higher than that (4.61) of the original PKA ligand. Although the mechanism of interaction between NOR and PKA needs to be confirmed by a future X-ray crystallographic study, these results suggest that NOR inhibits PKA more strongly than its ligand. According to the docking program, the binding residues that interact with NOR-S and NOR-R were GLU127, ASP184, and LYS72 of PKA (Fig. 9); these results indicate that NOR may inhibit PKA activity by binding at the active site of PKA.

In our previous studies, NOR was shown to promote VEGF-induced Notch1 activation and thereby showed an anti-migration activity [11], [12]. To clarify the interplay between the PKA pathway and the Notch1 pathway, the PKA inhibitor H-89 and the Notch1 inhibitor DAPT were used. The observation that H-89 improved Notch1 activation but DAPT failed to affect PKA activation suggested that PKA may act upstream of Notch1.

In summary, the present study demonstrated the crucial role of the PKA pathway in VEGF-induced endothelial cell migration. The inhibitory effect of NOR was attributed to its modulation of the PKA pathway, key points of which were the disruption of p65/IκBα complex and the formation of the PKAc/p65 complex. These results suggest that NOR inhibits VEGF-induced endothelial cell migration via a cAMP-PKA-NF-κB/Notch1 signaling pathway.
